# A multilevel analysis of the social determinants associated with symptoms of acute respiratory infection among preschool age children in Pakistan: A population-based survey

**DOI:** 10.1371/journal.pone.0260658

**Published:** 2021-12-16

**Authors:** Oluwafunmilade Deji-Abiodun, David Ferrandiz-Mont, Vinod Mishra, Chi Chiao

**Affiliations:** 1 Department of Medicine and Center for Global Health, University of Chicago, Chicago, IL, United States of America; 2 Public Health Surveillance and Emergency Response Department of Vallès Occidental and Vallès Oriental, Public Health Agency of Catalonia, Sant Cugat del Vallès, Barcelona, Spain; 3 United Nations Population Division, New York City, NY, United States of America; 4 Institute of Health and Welfare Policy, College of Medicine, National Yang Ming Chiao Tung University, Taipei, Taiwan; University of Western Australia, AUSTRALIA

## Abstract

**Background:**

As advocated by WHO in “C*losing the Health Gap in a Generation*”, dramatic differences in child health are closely linked to degrees of social disadvantage, both within and between communities. Nevertheless, research has not examined whether child health inequalities include, but are not confined to, worse acute respiratory infection (ARI) symptoms among the socioeconomic disadvantaged in Pakistan. In addition to such disadvantages as the child’s gender, maternal education, and household poverty, the present study also examined the linkages between the community environment and ARI symptoms among Pakistan children under five. Furthermore, we have assessed gender contingencies related to the aforementioned associations.

**Methods:**

Using data from the nationally representative 2017–2018 Pakistan Demographic and Health Survey, a total of 11,908 surviving preschool age children (0–59 months old) living in 561 communities were analyzed. We employed two-level multilevel logistic regressions to model the relationship between ARI symptoms and individual-level and community-level social factors.

**Results:**

The social factors at individual and community levels were found to be significantly associated with an increased risk of the child suffering from ARI symptoms. A particularly higher risk was observed among girls who resided in urban areas (AOR = 1.42; *p*<0.01) and who had a birth order of three or greater.

**Discussions:**

Our results underscore the need for socioeconomic interventions in Pakistan that are targeted at densely populated households and communities within urban areas, with a particular emphasis on out-migration, in order to improve unequal economic underdevelopment. This could be done by targeting improvements in socio-economic structures, including maternal education.

## Introduction

Acute respiratory infection (ARI) is known to be the leading cause of morbidity and mortality among young children under the age of 5 years [1]. Globally, over 1.23 million children die of pneumonia before reaching their 5th birthday, which is the equivalent of 141 child deaths per hour or 3,400 deaths per day. As of 2018, this single infectious disease contributes more than 800,000 deaths to the under-five mortality count [2]. Children living in countries that suffer from the disproportionate impacts of infectious diseases globally must now contend with the additional catastrophic impact of COVID-19; this pandemic has the potential to reverse decades of progress in protecting children from preventable diseases [3]. In 2020, a total of fifteen countries can be identified with the highest mortality due to ARIs among children aged under 5 years old. Children in Pakistan make up 16.6% of the global mortality due to ARI and diarrhea, with the country ranking third after India and Nigeria. The global community action plan, which aims at a 75% reduction in incidence of severe ARIs in children, is geared towards closing the gap by 2025 [2–4].

In view of the above, there is ongoing global concern about the influence of a specific country and that country’s contextual social determinants. This encompasses how these bring about health inequality within and between countries, and how individuals are subject to uneven systems of social imbalance that drive the poverty level and health systems that individuals live and work in [5]. Pakistan is the sixth most populous country (207 million) in the world with 64% of its population residing in rural areas [6]. Since its independence in 1947, the country has experienced countless inter-state and intra-state conflicts and is characterized as a volatile geopolitical region. In south Asia, Pakistan is ranked as the second lowest country in terms of progress according to the index of Sustainable Development Goals (SDGs) and this can be largely attributed to the sociopolitical instability of the country [7], which has resulted in 38% of the population living below the poverty line. Coupled with the weakened situation of the country, another systemic challenge is present and this is migration, which has long played a key role in shaping the size and distribution of Pakistan’s population.

Pakistan has been a destination for large numbers of cross-border migrants and refugees. The influx of these migrant groups, together with the increasing number of rural people being displaced by agricultural modernization and mechanization, have contributed to the substantial increase in the level of urbanization within the country, especially in the more industrialized provinces [8]. These complex and substantial movements have resulted in profound changes to the dynamics of the settlement patterns, and also in deep socioeconomic and cultural transformations, which have in turn created widening economic opportunities [8]. In scenarios with such gross inequalities, importantly preceded by the effects of sociopolitical instability within a given region, Pakistan has been the destination of large number of cross-border immigrants/refugees, as well as significant numbers of within country migrants [8].

This social instability has had numerous impacts on the country’s economic, health, and environmental systems and it is therefore imperative to understand how internal migration affects different aspects of the system, especially the health sector. The attainment of healthcare and a healthy lifestyle by individuals may be affected by cultural differences [8], including both social and financial constrains. These are influenced by various structural factors, such as household income, education level, social status, economic opportunities and the conditions in which a family lives [9]. The susceptibility of poor and vulnerable populations to their environment, specifically children under five years old, should be a priority in terms of the agenda outlined above, as it is a more enduring inequality problem. Therefore, scaling up equitable access to health determinants is a global concern because such social access plays a pivotal role in determining economic growth. The multiplier effect caused by contextual rapid urbanization contributes to the rising inaccessibility of populations to basic sociodemographic factors [10–18].

In addition to the distal factors that influence multi-level factors, proximal determinants, such as childhood ARI, act at the household level where the children are embedded; these are often closely related to the behavior and capacity of the household’s socioeconomic status (SES), namely maternal education, fertility preferences (including birth order), choice of cooking fuel, women’s socioeconomic autonomy, and urban/rural residence [19]. When such a context shapes health inequalities, this drives vulnerable population, namely pre-school children under 5 years old, into a situation where they are subject to the social environment in which they find themselves. These effects occur in parallel with any systems put in place to deal with their vulnerability to health problems.

Accordingly, the present study specifies social determinants at multiple levels; these include community-level characteristics, such as community education and community migration status, as well as household-level characteristics, namely maternal education, household poverty, household indoor cooking, household migration and residence. In addition to child’s characteristics, prior studies also suggested gender contingency, in which being female gendered may result in exposure to different risks due to biosocial factors [20]. The SES vulnerability of these children is hypothesized to increase the risk of ARI symptoms, but this has been studied only rarely in Pakistan. This study thus examines the linkages between social determinants and a child’s ARI symptoms, with a focus on both the individual as well as the community level. Furthermore, we explore the extent to which these ARI symptoms are related to the child’s gender, which in turn may be a consequence of gender inequality.

## Methods

### Data and study population

The present study employed a nationally representative dataset from the 2017–2018 Pakistan Demographic and Health Survey (PDHS). The data included interviews of ever-married women aged 15–49 years old and collected a wide range of information about them and their children, including socio-economic characteristics, migration related features and health factors, such as the acute respiratory infection symptoms of their children. The PDHS data was collected under strict ethical standards supervised by the ethical review boards of Macro International Inc. (a U.S.-based company that provides technical assistance to DHS surveys worldwide), and other relevant implementing partners. Furthermore, informed consent was obtained from each of the respondents before conducting the surveys. The PDHS datasets are released for public use and can be applied for research use after approval by MEASURE DHS (https://dhsprogram.com/). The Ethical Committee of National Yang Ming Chiao Tung University approved the study protocol (IRB Number: YM106042E-4).

DHS data was collected by a stratified two-stage sampling strategy [21]. Stratification was performed by separating each of the eight regions that comprised Pakistan (Punjab, Sindh, Khyber Pakhtunkhwa, Balochistan, Islamabad, Gilgit Baltistan, Azad Jammu and Kashmir, and FATA) into urban and rural areas. Then, two-stage selection was carried out independently for each stratum. Further information on the DHS can be found at www.measuredhs.com. This study focused on 11,989 surviving children under the age of 5 years old. After exclusion of 81 pre-school children who lacked information about ARI symptoms and some other major explanatory variables, the analytical sample consisted of 11,908 surviving children under 5 years old living in 561 clusters.

### Measures

#### Outcome measure

Symptoms among the children regarding acute respiratory infection (ARI) were assessed based on the mothers’ reports, specifically whether or not their children had been ill with a cough accompanied by short, rapid breathing that was chest-related, and/or had had difficulty in breathing that was chest-related; the time period examined was the two weeks preceding the DHS children survey. Responses were separated into two categories. Where young children had suffered from ARI symptoms, this is coded as Y = 1 or “yes” and where young children not suffered from ARI symptoms, this is coded Y = 0 or “no”.

The *community measures* used by the DHS household survey consisted of: (1) community out-migration due to economic betterment, which was computed by averaging households who had a member aged 18–59 who had out-migrated for work due to economic difficulties and measuring the proportion of such households within the community during the past 12 months period preceding the survey; (2) community in-migration, which was computed by assessing the proportion of in-migration households within the community; and (3) community education level, which was assessed as the percent of community members within a community block who had received no education.

The measures used in the PDHS to assess household characteristics included *household poverty*, *household indoor cooking*, *household immigration status*, *household left-behind status and urban residence*. *Household poverty* was derived from the standardized household wealth index, namely five quintiles of asset-based measurements for the country [22]. Household poverty refers to household wealth below 40% at the national level; that is, a household was coded as being in poverty if it was within the lower two quintiles. The analysis assessed household migration by measuring the immigration status and the left-behind status of households. Household immigration status refers to whether a household member had an immigrant member. (yes/no). Household left-behind status was measured by several questions, including age and reason for migration of each household member during the past 12-months period preceding the survey. Left-behind households were identified as a household that had experienced labour migration of a household member, that is a person who had migrated for work in order to seek economic betterment during the past 12 months, and who was aged older than 18 and younger than 60.

In addition to household characteristics, the present study also included maternal education (no education, incomplete primary education, complete primary education, incomplete secondary education, complete secondary education and higher than secondary education), as well as various characteristics of the children, such as gender, birth weight (low birth weight, normal birth weight and unknown), age (less than 1 years old, 1–2 years old, 3–5 years old), birth order (first to the third, fourth to sixth, and after the sixth) and being an orphan.

### Analytical strategy

All the statistical analyses were conducted by Stata 15.0 [23] and weighted to adjust for the sample design. The analytical strategy consisted of two parts. The first part consisted of descriptive tabulation that characterized the distribution of the community-level and individual-level social factors of the young children by gender. Given that children within a given community cluster are likely to exposure to a somewhat similar social effect, this means that children from the same community often share similar lifestyles, and these behaviors may differ from their counterparts in other community clusters. Accordingly, the second part involved multilevel logistic regression models using the *melogit* command in Stata 15.0 [23]. In order to estimate the community influence, we calculated the intra-class correlation coefficient (ICC), which represents the percentage of the total variance related to experiencing ARI symptoms across communities [24]. The ICC from the intercept-only model was 0.16 (*p* < 0.01), suggesting that 16% of the total variation in child’s ARI symptoms were explained by community clusters where these children resided. As a result, we employed a two-level multi-level logistic regression models where the children were at level 1 and these children were then nested within their communities at level 2.

To assess the effects of social determinants on the symptoms of ARI, analytical progressive adjustments were conducted. The first one used a crude model in which we studied the association of each covariate with the likelihood of ARI symptoms. Then, we included the individual-level factors in the model in order to determine their independent influence on the children’s ARI symptoms (Model 1). Next, we added the community-level factors to Model 1 to disentangle the effects of social risks at each level. Given that one aim of the present study is to explore the contingent effects of child’s gender jointly with other individual characteristics on suffering from ARI symptoms, interactions terms were added to Model 3. The Model 3 specifically tested whether or not the association between a child’s gender and the presence of ARI symptoms depends on birth order and urban residence.

## Results

Table 1 shows the distribution of household and socio-demographic characteristics of the pre-school children by gender. Overall, 13.24% of the study sample presented with ARI symptoms with there being a higher prevalence among pre-school males compared to pre-school females (13.56% *vs*. 12.93%). More than 40% of the children came from poor households and almost half of these households practice indoor cooking. Near 3% of the children had a family member who had immigrated and more than 15% had a relative in their household who out-migrated for economic reasons. When comparing males and females, females were more often resident in a poor household (42.67% *vs*. 41.35%), and a slightly lower proportion of them had a family member who immigrated or out-migrated. Finally, children with an urban residence accounted for almost one third of the sample, with a higher proportion of boys living in urban areas compared to girls (32.84% *vs*. 31.77%).

**Table 1:** Sample characteristics and distributions among pre-school females and males (age 0–5), 2017–2018 Pakistan demographic and health survey.

		Total	Female	Male
Prevalence of ARI symptoms (%)		13.24	12.93	13.56
***Household characteristics***				
Household poverty		42.01	42.67	41.35
Household indoor cooking		49.90	49.97	49.82
Household immigration status				
	Household with an immigration member	2.77	2.55	2.98
Household left-behind status				
	Household with a migrant member due to economic better-off	15.63	15.43	15.84
Urban residence		32.30	31.77	32.84
***Maternal characteristics***				
Maternal education (%)				
	No education	48.44	48.18	48.71
	Incomplete primary education	5.54	5.11	5.98
	Complete primary education	10.94	11.66	10.21
	Incomplete secondary education	11.23	10.46	12.01
	Complete secondary education	10.74	11.06	10.41
	Higher than secondary education	13.11	13.54	12.67
***Child’s characteristics***				
Gender (%)				
	Male	49.72		
	Female	50.28		
Birth weight (%)				
	Low birth weight (< 2,500 grams)	3.57	3.31	3.83
	Normal weight	12.95	13.31	12.58
	Unknown	83.49	83.39	83.59
Age (Mean = 2.00; Std Dev = 1.42)				
	Less than 1 year old (%)	20.05	20.10	20.00
	1–2 years old (%)	39.77	39.44	40.11
	3–5 years old (%)	40.18	40.47	39.89
Birth order (%)				
	1–3	63.27	63.42	63.12
	4–6	28.30	27.91	28.70
	>6	8.43	8.67	8.18
Being orphan (%)		0.96	0.98	0.94
N		11,908	5,837	6071

*Note*: Percentages were weighted using individual-level sampling weights. Total *Ns* were unweighted. Percentages may not add up to 100 due to rounding.

In terms of maternal characteristics, half of the mothers had not attended any type of school and only 13% of the mothers had attained an education level higher than secondary education. A higher proportion of mothers of female children had pursued studies beyond secondary education compared to mothers with male children (13.54% *vs*. 12.67%). Turning to birth characteristics, 3.57% of the preschool children had experienced a low birth weight with a slightly higher proportion of males having this compared to females (3.83% *vs*. 3.31%). Almost two-thirds (63.27%) are first to third born children, followed by 28.30% with a birth order from 4 to 6 and less than 10% with a birth order higher than 6. A higher proportion of males compared to females are the 4th to 6th child of the family (28.70% *vs*. 27.91%).

Table 2 presents the two-level logistic regression models used to investigate the relationship between social and health factors in relation to ARI symptoms. The crude model shows the single effects of community, household, maternal and child characteristics on ARI symptoms. Under 5 years-old children living in communities with a high proportion of individuals that had out-migrated due to economic reasons are more prone to experience ARI symptoms (OR = 3.47; *p* < 0.01) whereas those children living in communities with a high prevalence of people who immigrated are less likely to experience these symptoms (OR = 0.46; *p* < 0.05). The odds of suffering from ARI symptoms were higher among boys and girls coming from deprived households (OR = 1.30; p < 0.01) and households where they practised indoor cooking (OR = 1.24; *p* < 0.01). By way of contrast, those children with mothers who had pursued an education higher than secondary were less likely to experience ARI symptoms (OR = 0.69; *p* < 0.01). Turning to the characteristics of the children, girls had lower odds of experiencing ARI than boys (OR = 0.85; *p* < 0.01) and the risk of suffering ARI symptoms decreased with age (OR = 0.90; *p* < 0.01). Children with a low birth weight were more prone to have ARI symptoms compare with those with a normal birth weight (OR = 1.66; *p* < 0.01), and children with birth order from 4 to 6 were more likely to experience ARI symptoms than those with a lower birth order (OR = 1.17; *p* < 0.05).

**Table 2:** Multilevel regression models of community influences on the likelihood of symptoms of acute respiratory infection among pre-school children in Pakistan, DHS 2017–2018.

		AOR (95% CI)							
		Crude Model		Model 1		Model 2		Model 3	
***Household characteristics***									
Household poverty		1.30	(1.13, 1.48)**	1.25	(1.06, 1.47)**	1.24	(1.05, 1.46)*	1.24	(1.05, 1.47)*
Household indoor cooking		1.24	(1.08, 1.42)**	1.14	(0.98, 1.34)	1.89	(0.93, 1.27)	1.08	(0.92, 1.27)
Household immigration status (ref = No)									
	Household with an immigration member	0.84	(0.62, 1.13)	0.90	(0.67, 1.22)	0.92	(0.68, 1.26)	0.92	(0.68, 1.26)
Household left-behind status (ref = No)									
	Household with a migrant member due to economic better-off	1.02	(0.87, 1.19)	1.01	(0.86, 1.19)	0.94	(0.80, 1.11)	0.95	(0.80, 1.12)
Urban residence (ref = No)		0.93	(0.78, 1.11)	1.12	(0.92, 1.37)	1.23	(0.99, 1.53)	1.05	(0.82, 1.33)
***Maternal characteristics***									
	Incomplete primary education	1.28	(0.99, 1.64)	1.31	(1.02, 1.69)*	1.31	(1.02, 1.70)*	1.30	(1.01, 1.68)*
	Complete primary education	1.04	(0.85, 1.28)	1.10	(0.89, 1.36)	1.10	(0.89, 1.36)	1.10	(0.89, 1.36)
	Incomplete secondary education	1.09	(0.90, 1.32)	1.17	(0.95, 1.43)	1.16	(0.95, 1.43)	1.17	(0.95, 1.43)
	Complete secondary education	0.97	(0.80, 1.19)	1.06	(0.86, 1.32)	1.07	(0.85, 1.33)	1.06	(0.85, 1.32)
	Higher than secondary education	0.69	(0.56, 0.83)**	0.76	(0.61, 0.95)*	0.76	(0.61, 0.96)*	0.76	(0.61, 0.96)*
***Child’s characteristics***									
Birth weight (ref = Normal)									
	Low birth weight (< 2,500 grams)	1.66	(1.21, 2.27)**	1.53	(1.12, 2.10)**	1.52	(1.11, 2.09)**	1.51	(1.10, 2.07) *
	Unknown	1.08	(0.90, 1.28)	0.94	(0.78, 1.14)	0.92	(0.76, 1.11)	0.92	(0.76, 1.11)
Age (in years)		0.90	(0.87, 0.94)**	0.90	(0.87, 0.94)**	0.90	(0.87, 0.94)**	0.90	(0.87, 0.94)**
Birth order (ref = 1–3)									
	4–6	1.17	(1.04, 1.33)*	1.14	(1.00, 1.29)	1.13	(1.00, 1.29)	0.99	(0.83, 1.17)
	>6	1.17	(0.97, 1.41)	1.12	(0.92, 1.36)	1.12	(0.92, 1.36)	0.92	(0.70, 1.20)
Orphan status (ref = No)		1.40	(0.78, 2.54)	1.45	(0.80, 2.64)	1.45	(0.79, 2.63)		
***Community characteristics***									
Community out-migration due to economic better-off		3.47	(1.75, 6.88)**			3.37	(1.58, 7.17)**	3.40	(1.59, 7.26)**
Community im-migration		0.46	(0.25, 0.84)*			0.71	(0.35, 1.44)	0.70	(0.35, 1.41)
Community education		1.54	(0.96, 2.48)			1.11	(0.59, 2.07)	1.11	(0.60, 2.08)
***Gender interaction***									
Female gender (ref = Male)		0.85	(0.76, 0.95)**	0.86	(0.77, 0.96)**	0.86	(0.77, 0.96)**	0.65	(0.54, 0.77)**
	Female × Birth order (4–6)							1.34	(1.04, 1.71)*
	Female × Birth order (> 6)							1.50	(1.04, 2.18)*
	Female × Urban residence							1.42	(1.14, 1.78)**

Note

*p<0.05

**p<0.01

Model 1 is an adjusted model that attempts to disentangle the individual effects of household, maternal and child characteristics on ARI symptoms. Household poverty remains significantly associated with ARI symptoms (AOR = 1.25; *p* < 0.01) while the association with household indoor cooking become no longer significant. The odds of having ARI symptoms were decreased among young children with a mother who had an education level higher than secondary education (AOR = 0.76; *p* < 0.05) and increased among those with a mother that have not completed primary education (AOR = 1.02; p < 0.05). A low birth weight and a lower age remained risk factors for ARI symptoms (AOR = 1.53; *p* < 0.01 and AOR = 0.90; *p* < 0.01, respectively), while female gender remained a protective factor (AOR = 0.86; *p* < 0.01).

Model 2 incorporates community level variables to reveal the community influences on ARI symptoms. Communities with a high prevalence of individuals that had out-migrated due to economic reasons had this to be a risk factor for ARI symptoms (AOR = 3.37; *p* < 0.01). After the inclusion of community variables in the model, household poverty continued to be consistently associated with ARI symptoms, but the significance of the association decreased; the remaining variables did not result in any remarkable changes in the intensities and directions of the various associations.

Model 3 adds interaction terms to the model related to the sex of the child, the birth order and urban residence in order to test for variations in the effects on ARI symptoms between girls and boys, for differences in birth orders and for differences in urban-rural residence. For example, as in all previous models, the results shown that pre-school females are less prone to experience ARI symptoms than their male counterparts are. However, when comparing males and females living in urban areas, the likelihood of females suffering from ARI is 0.92 times higher than their male counterparts (0.65 x 1.42 = 0.92). Likewise, when taking into account the birth order, our analysis revealed that the odds of having ARI symptoms among females is higher than among males when their birth order is either between 4 to 6 or is higher than 6 (where the effect are 0.87 and 0.97, respectively).

## Discussion

We used multilevel modelling to understand the interplay of the social determinants of ARI symptoms among preschool children in Pakistan that occurs between the community-level factors and the individual social backgrounds that they are embedded in. This study addressed gaps in the literature by examining the linkages between social determinants and risk of a child developing ARI symptoms, as well as further investigating the effect of community influences on such relationships; this was done by incorporating the presence of in-migration factors at both levels, which may affected the variation in ARI symptoms [25].

Supported by the hypothesis of social determinants [26], a high community-level prevalence of adults who had out-migrated due to economic reasons was revealed to be linked to a higher risk of ARI symptoms. A common reason of out-migration is the household’s economic poverty status, as poor households might be expected to consider out-migration in order to explore better economic opportunities with the migrant often being the head of households or the main bread winner [27]. Typically, out-migration of the bread winner subjects the dependent household members to a significant economic disadvantage for a period of time [28]. We have conducted additional analysis to tabulate the presence of child’s ARI symptoms by whether or not out-migrants sent back remittances. Among the study sample, 8.14% came from households with out-migrants who sent back remittances. Yet the prevalence of ARI symptoms among these children (13.70%) was similar to the ARI risk among the other children (13.20%).

Our results are consistent with prior research showing that a higher risk of ARI symptoms is significantly associated with household poverty [29–31]. The level of maternal education is an indicator of household economic status as this determinant reduces the risk of ARI symptoms in young children [32, 33]. At the community level, the effect of migration of the male heads of household on dependants is very likely to contribute to household poverty and thus bring about an increase in social vulnerability [28]. Linked with community out-migration, there would most likely be a drastic reduction in social and economic development in such communities that have significant numbers of heads of household who have migrated; such migration will reduce the institutions that improve the social determinants of the residents.

Being female gendered seems to be a protective factor in line with prior studies. The reason for this gender difference in ARI symptoms seems to be differences in lung structure and function between the genders, in that males have larger lungs and females attain an earlier maturity and long-term airway patency compared to their male counterparts [20]. In addition, children born at a low birth weight [33] and those who were younger [34], both had a higher risk of ARI symptoms. Nevertheless, the significant association that is found for gender, birthweight, and age of children did not result in remarkable changes with respect to ARI symptoms in terms of the association’s intensity and direction after the inclusion of community and household characteristics.

Interestingly, our analyses did not find residential differences affected the likelihood of ARI symptoms among young children. The lack of an effect for residence may be because the social forces that elicit residential disparities in terms of lifestyle pattern and SES minority may not be important here. We conducted residence stratification to examine rural/urban differences in indoor house cooking and out-migration. This additional analysis indicated that both of them were more common in rural settings. Yet our progressive adjustments from Crude Model to Model 1 suggest that household indoor cooking became non-significant when household poverty was significantly associated with a greater risk of ARI symptoms.

Our findings should be interpreted within the context of the study’s limitations. In addition to the common limitations associated with ARI symptoms being a self-reported measure, some acute respiratory infections are self-limiting and can easily be ignored by mothers, especially when a child is immunocompromised and living in a less vulnerable community. Thus, low level ARIs may have been excluded from the current study. Even though we intended to include childhood vaccinations in our models, this was problematic because there were about 75% of the participants with an unknown status or/and missing responses regarding this variable. In addition, the possibility of recall bias needs to be taken into account for birth weight status, which was provided by the mother of the child, and had to be recalled from the time of birth.

Next, due to data limitations, some factors, such as poverty, are broad-based and dichotomously represented in this study; while such an approach is frequently used, it can lead to the study missing variation in associations that exists across the continuum from poverty to affluence. However, by using a multilevel analytical approach, our study does provide important insights and has identified various multilevel social determinants of childhood ARI symptoms in Pakistan. Future in-depth research is needed to gain a better understanding of the health impact of migration due economic pressure (both in and out migration) in Pakistan on vulnerable dependants, these include pre-school children and the women who are left as the head of households and/or the household bread winner. Such migration means that dependants are left with no one who is socially responsible for them or that might help them to gain a better understanding of childhood illnesses such as ARI. Our findings will benefit policy makers by allowing them to develop community-level and related socioeconomic interventions that specifically target densely populated households and communities characterized by systemic problems such as out-migration. Such interventions ought to improve the level of unequal economic development. This might be done by accessing improved general socio-economic structures, such as maternal education and health care services.
